# Environmental contamination with carbapenem resistant *Acinetobacter baumannii* in healthcare settings in Fiji: a potential source of infection

**DOI:** 10.3389/fcimb.2024.1429443

**Published:** 2024-09-23

**Authors:** Sakiusa C. Baleivanualala, Silivia Matanitobua, Yvette Samisoni, Vika Soqo, Shayal Smita, Josese Mailulu, Ilisapeci Nabose, Alvina Lata, Christina Shayam, Radhika Sharma, Donald Wilson, John A. Crump, James E. Ussher

**Affiliations:** ^1^ Department of Microbiology and Immunology, School of Biomedical Sciences, University of Otago, Dunedin, New Zealand; ^2^ College of Medicine, Nursing and Health Science, Fiji National University, Suva, Fiji; ^3^ Maurice Wilkins Centre for Molecular Biodiscovery, University of Auckland, Auckland, New Zealand; ^4^ Fiji Centre for Disease Control, Ministry of Health and Medical Services, Suva, Fiji; ^5^ Department of Infection Prevention and Control, Aspen Medical, Lautoka Hospital, Lautoka, Fiji; ^6^ Microbiology Laboratory, Aspen Medical, Lautoka Hospital, Lautoka, Fiji; ^7^ Microbiology Laboratory, Labasa Hospital, Ministry of Health and Medical Services, Labasa, Fiji; ^8^ Pacific Laboratory Specialists, Suva, Fiji; ^9^ Department of Infection Prevention and Control, Colonial War Memorial Hospital, Ministry of Health and Medical Services, Suva, Fiji; ^10^ Department of Infection Prevention and Control, Labasa Hospital, Ministry of Health and Medical Services, Labasa, Fiji; ^11^ Centre for International Health, Division of Health Sciences, University of Otago, Dunedin, New Zealand; ^12^ Otago Global Health Institute, University of Otago, Dunedin, New Zealand; ^13^ Department of Microbiology, Awanui Labs, Dunedin Hospital, Dunedin, New Zealand

**Keywords:** carbapenems, antimicrobial resistance, *Acinetobacter baumannii*, Fiji, hospital environment, hospital-acquired infection

## Abstract

**Introduction:**

There are multiple ongoing outbreaks of carbapenem resistant *Acinetobacter baumannii* (CR*Ab*) infection in Fiji’s hospitals. CR*Ab* is able to colonize and persist on various hospital surfaces for extended periods. We conducted a study to understand the extent of hospital environmental contamination and phylogenetic links with clinical isolates.

**Methods:**

Swabs were collected from high-touch surfaces at Colonial War Memorial Hospital (CWMH) September 2021 and December 2022; Lautoka Hospital (LTKH) August 2022; and Labasa Hospital (LBSH) November 2022. All bacterial isolates were identified, and antimicrobial susceptibility testing (AST) performed; isolates resistant to carbapenems and producing a carbapenemase underwent whole genome sequencing. Comparison was made to clinical isolates obtained from CWMH in 2016–2017 and 2019–2021 and from LTKH and LBSH from 2020–2021.

**Results:**

From the 180 environmental samples collected, ten (5.6%) CR*Ab* were isolated; no other carbapenem-resistant gram-negative organisms were isolated. Seven (70%) of the CR*Ab* were isolated from CWMH and three (30%) from LTKH; no CR*Ab* were isolated from LBSH. Of the seven CWMH CR*Ab*, two were sequence type 2 (ST2), three ST25, and two ST499. All LTKH isolates were ST499. The two environmental CR*Ab* ST2 isolates were closely genetically linked to isolates obtained from patients in CWMH, LTKH, and LBSH 2020–2021. Similarly, the three environmental CR*Ab* ST25 isolates were closely genetically linked to isolates obtained from patients admitted to CWMH in 2019–2021 and LBSH in 2020. The environmental CR*Ab* ST499 isolates represented two distinct clones, with clone 1 comprising two genetically identical isolates from CWMH and clone 2 the three isolates from LTKH. Although no genetic linkages were observed when comparing environmental ST499 isolates to those from CWMH patients in 2020–2021, both clone 1 isolates were genetically identical to an isolate obtained from a patient admitted during the sampling period.

**Conclusion:**

Our study highlights the contamination of high-touch surfaces within Fiji hospitals with CR*Ab*, suggesting that these may serve as important sources for CR*Ab*. Phylogenetic linkages to CR*Ab* isolated from patients since 2019 underscores the persistence of this resistant pathogen in hospital settings and the ongoing risk for hospital-acquired infections.

## Introduction

*Acinetobacter baumannii* is a gram-negative opportunistic pathogen known for its remarkable ability to thrive in hospital environments, where it can survive on invasive devices, dry surfaces, and even human skin for extended periods (Wendt et al., 1997; Dijkshoorn et al., 2007; Marchaim et al., 2007; Katzenberger et al., 2021). It exhibits intrinsic resistance to desiccation and disinfectants, and has the ability to resist various classes of antimicrobials, including carbapenems, contributing to heightened morbidity and increased mortality (Peleg et al., 2008). The emergence of carbapenem-resistant *A. baumannii* (CR*Ab*) presents a substantial public health concern, challenging the efficacy of carbapenems that serve as one of the last resorts in antimicrobial treatment (Malchione et al., 2019). While community-acquired infections of CR*Ab* have been reported, hospital-acquired infections are much more common and pose a significantly higher risk due to their association with severe cases of pneumonia, bloodstream infections, urinary tract infections, and also higher mortality rates, particularly in vulnerable patients with weakened immune systems (Munoz-Price and Weinstein, 2008; Kurihara et al., 2020).

Contamination of medical equipment and surfaces in healthcare settings, especially in hospitals and long-term care facilities, significantly contributes to the spread of antimicrobial resistant (AMR) pathogens, including CR*Ab*, intensifying the risk for vulnerable patients and contributing to the incidence of hospital-acquired infections (Hanczvikkel and Tóth, 2018; Saliba et al., 2021). These resistant pathogens can be transmitted through both direct and indirect hand contact with hard, non-porous surfaces, such as high touch equipment and surfaces including trolleys, bedside tables, bed rails, bed linens, curtains, and floors, leading to persistent outbreaks in hospital settings (Boyce, 2007; Musoke et al., 2021). Cross-transmission via the hands of health care workers (HCWs), which have become contaminated either directly from patient contact or indirectly by touching contaminated environmental surfaces, has been implicated in 20 to 40% of HAIs (Weber et al., 2010; Olmsted, 2016). Moreover, transmission of antimicrobial resistant (AMR) pathogens between non-HCWs and patients has also been reported (Mboowa et al., 2021). This underscores the critical need for stringent hygiene practices and regular disinfection protocols to prevent the spread of AMR pathogens and infections. Moreover, inadequate sterilization of medical equipment and suboptimal environmental disinfection contribute to the survival of CR*Ab*, while the persistence of biofilms in plumbing systems further contributes to their survival and multiplication (Fahy et al., 2023).

Several studies have highlighted the genetic similarity between strains isolated from patients, HCWs, and the hospital environment, indicating a close interconnection in the transmission network of AMR pathogens within healthcare settings (Zhang et al., 2022). This interconnectedness highlights the significant interplay in the transmission of pathogens within healthcare facilities, challenging our traditional understanding of the concepts of reservoirs and sources. Reservoirs are typically environments or hosts where pathogens are maintained and multiply, and sources are specific points from which the pathogen is transmitted to a new host identified through specific interactions or events at distinct times and locations that facilitate the spread of the pathogen (Haydon et al., 2002). Pathogens can circulate among patients, HCWs, and the environment, making it challenging to pinpoint specific sources of transmission. Of note, the difficulty in identifying the directionality of AMR outbreaks in hospitals has mainly been due to lack of source identification (Zimmerman et al., 2017; Baleivanualala et al., 2023). Several studies have also highlighted the ongoing presence of pathogens on hands of HCWs, in the hospital environment, and on medical equipment, suggesting the importance of identifying the sources of AMR pathogens (Musa et al., 1990; Pittet et al., 2006; Wolfensberger et al., 2018). While effective hand hygiene has demonstrated success in preventing many hospital-acquired infections, the role of environmental cleaning practices and microbial decontamination of surfaces in influencing the transmission of AMR pathogens cannot be overlooked (Pittet et al., 2006; Dettenkofer and Spencer, 2007; Herrera Villanueva, 2020; Parry et al., 2022). Investigations consistently reveal the presence of AMR pathogens on various surfaces within hospitals, requiring a proactive approach such as environmental swabbing to enhance surveillance efforts to monitor and mitigate the spread of AMR infections in healthcare settings (Acolatse et al., 2022; Nygren et al., 2023). Therefore, molecular epidemiological investigations are important in identifying potential infection sources.

In the heart of the South Pacific, Fiji, an upper-middle-income country, is confronting the escalating threat of AMR, particularly with CR*Ab* (Zimmerman et al., 2017; Baleivanualala et al., 2023). *Acinetobacter baumannii* international high risk clone sequence type 2 (ST2) (Shelenkov et al., 2023), is known for its worldwide prevalence and the high mortality associated with infection. This strain was first detected in Fiji in 2016 during an outbreak in the neonatal intensive care unit (NICU) of Fiji’s largest hospital, the Colonial War Memorial hospital (Wendt et al., 1997; Peleg et al., 2008; Zimmerman et al., 2017; Baleivanualala et al., 2023). The ability of CR*Ab* to colonize hospital environments, including invasive devices, dry surfaces, and human skin for extended periods, has made them potential sources of transmission (Wendt et al., 1997). However, the sources of this outbreak strain have not been definitively determined. Phylogenetic analysis has linked this outbreak strain to others isolated from Samoa, New Zealand, and Australia, suggesting transnational spread (Baleivanualala et al., 2023). Moreover, this NICU outbreak clone has persisted, with two additional ST2 clones identified in 2019 and 2020, as well as two clones each of ST25 and ST499 (Baleivanualala et al., 2023, 2024). These strains harbor carbapenem resistant gene *bla*_OXA-23_, with or without *bla*_NDM-1_, conferring resistance to carbapenem antimicrobials (Baleivanualala et al., 2023, 2024). Ongoing transmission of these CR*Ab* strains has been detected within and between the different Fijian hospitals, highlighting the infection control challenge posed by such strains (Baleivanualala et al., 2023, 2024; Zimmerman et al., 2017). Furthermore, there is evidence of transmission to other countries in the region (Zimmerman et al., 2017; Baleivanualala et al., 2023). Suboptimal hand hygiene practices and the reuse of single-use equipment, as identified in a previous CR*Ab* outbreak report in Fiji, (Zimmerman et al., 2017) may have contributed to these outbreaks of hospital-acquired infection. Given Fiji’s central role as an economic, educational, and technological hub in the South Pacific and its position as a potential hotspot of CR*Ab* in the Oceania region (Baleivanualala et al., 2023, 2024; Zimmerman et al., 2017) it is crucial to identify the sources of CR*Ab* within Fiji’s healthcare settings to inform targeted effective infection control measures to mitigate and control their spread. In this study, we conducted a molecular epidemiological analysis to investigate the extent of environmental contamination and the phylogenetic relationships among CR*Ab* isolates from high-touch surfaces and medical equipment in the three major hospitals in Fiji.

## Materials and methods

### Study design and setting

We undertook a molecular epidemiology investigation of CR*Ab* obtained from high-touch surfaces (environment and medical equipment) in intensive care units (ICUs; adult, maternity, pediatrics and neonatal), and other medical and surgical inpatient settings across the three major hospitals in Fiji: the Colonial War Memorial Hospital (CWMH), Lautoka Hospital (LTKH), and Labasa Hospital (LBSH) (see map, **Supplementary Figure 1**). Subsequently, phylogenetic analyses were employed to assess the genetic relationships between CR*Ab* obtained from high-touch surface samples and CR*Ab* isolated from patients over the previous two years. Our sampling strategy was purposive and specifically focused on hospital settings known to have a higher prevalence and ongoing transmission of carbapenem resistant organisms since 2016 (Zimmerman et al., 2017; Baleivanualala et al., 2023). This targeted approach prioritized areas within the hospitals that are involved in intensive care, such as ICUs and surgical units, due to their higher risk of hospital-acquired infections and the increased vulnerability of patients in these settings. The selection of specific sites for swabbing and the number of samples collected was also guided by recommendations of the hospitals’ infection control teams, who used their professional judgment and experience from previous outbreaks to identify high risk areas; the number of samples collected from each hospital was not pre-specified. While the three hospitals are expected to adhere to similar infection prevention and control (IPC) guidelines, it is acknowledged that there may be variations in the implementation of these protocols due to resource availability, staff training, and hospital infrastructure.

### Sample acquisition

A total of 180 samples were collected from high-touch surfaces, which also include medical equipment (**Supplementary Table 1**). These were collected from the CWMH on two different occasions (26 September 2021, 1 December 2022) and from the LTKH and LBSH on a single occasion (29 August and 5 November 2022, respectively); the additional sampling at CWMH was conducted because it is the largest healthcare facility in Fiji. High-touch surfaces, recognized as frequently handled by both patients and HCWs, were identified and listed by local IPC officers in each hospital. This process was guided by both the IPC officer’s understanding of the hospital environment and those commonly reported as potential sources of contamination (Huslage et al., 2010; Cobrado et al., 2017). The sampling sites were based on: a) surfaces frequently handled by HCWs, patients, and visitors, such as bed railings, trolleys, and medical equipment, due to their high risk of harboring and transmitting pathogens; b) surfaces with direct clinical significance, such as ventilators and infusion pumps, having direct patient contact; c) surfaces or areas that are prone to contamination, such as sinks, door handles, and common workstations, that may lead to indirect patient contact.

The list of sampling sites can be found in the **Supplementary Table 1**.

Convenience sampling was employed via indirect sampling (**Supplementary Pages 1**–**3**) via swabbing of high touch surfaces using a moistened swab, rather than direct testing by the contact plate method or using a dry swab. This method was selected due to its greater efficacy in collecting samples from uneven and textured surfaces which are common in hospital settings and crucial for microbial surveillance. Moreover, it also offers simplicity and affordability that is suitable to the local context in Fiji. Additionally, indirect swabbing is noted for yielding higher sample volumes compared to the direct sampling method with dry swabs, enhancing the reliability of our results (Landers et al., 2010; Rawlinson et al., 2019; Jones et al., 2020). Sampling was done without prior notification of HCWs. Sampling was performed with a sterile swab (Copan, Murrieta, California, USA) moistened with tryptic soy broth (TSB) (Becton Dickinson, Sparks, USA) to increase microbial recovery (Landers et al., 2010). The moistened swab was gently rubbed onto specified locations covering an area of approximately 50cm^2^, employing horizontal, vertical, and diagonal motions (Kwan et al., 2011). The procedure was guided by the provided standard operating procedure and carried out by both the IPC team and laboratory scientists, who swabbed at least 50 cm² on various surfaces without the aid of a template. Their approach also relied on professional judgment to estimate the area, ensuring flexibility and adaptability to each surface’s unique characteristics. Although this method allows for adaptability, it also introduces some variability into the sampling process.

### Bacterial cultivation

Collected samples underwent processing in the microbiology laboratories of LTKH and LBSH, whereas samples from CWMH were processed at the Medical Teaching Laboratory of the College of Medicine, Nursing, and Health Science, Fiji National University. Swabs were inoculated into the TSB vials and incubated at 37°C for 24 hours aerobically. This culture was then sub-cultured onto MacConkey agar (Becton, Dickinson, Sparks, Nevada, USA), a selective culture agar for gram-negative organisms (Becton Dickinson, 2013). Following overnight incubation, all resulting colonies were swabbed, and the swab placed into charcoal Amies transport medium (Copan, Murrieta, California, USA), prior to shipping to the University of Otago, Dunedin, New Zealand for further analysis. Upon receipt in New Zealand, swabs were inoculated on to commercially prepared sheep blood agar (Fort Richard Laboratories, New Zealand) and colonies identified as below.

### Isolate identification, and antimicrobial susceptibility testing

All isolates were identified using matrix-assisted laser desorption ionization-time of flight (MALDI-TOF) mass spectrometry (Biotyper version 4.0; Bruker Daltonics, Billerica, MA, USA). All organisms were tested for antimicrobial susceptibility testing (AST) following the European Committee on Antimicrobial Susceptibility Testing (EUCAST) guidelines using the disk diffusion method (**Supplementary Table 2**). The minimum inhibitory concentration (MIC) of meropenem was determined using the E-test (bioMérieux) while the MIC of colistin was established through the broth microdilution method (Liofilchem), following EUCAST recommendations (EUCAST, 2020). All carbapenem resistant isolates underwent screening for carbapenemase production using the adjusted modified carbapenemase inactivation method (AmCIM) (Nguyen Vu et al., 2020). Positive AmCIM isolates were subjected to whole genome sequencing (WGS).

### Genomic DNA extraction and whole genome sequencing

Genomic DNA extraction from overnight cultures was performed using the NucleoSpin^®^ Tissue kit (MACHEREY-NAGEL, Düren, Germany) following the manufacturer’s instructions (**Supplementary Page 4**). Subsequently, the DNA samples were submitted to Otago Genomics (Dunedin, New Zealand) for library preparation and sequencing. The libraries were prepared using the Illumina DNA Prep (M) Tagmentation kit and protocol (Illumina, San Diago, California, USA). Each of the libraries was assigned a unique index and underwent validation in accordance with Illumina’s protocol. Subsequently, the libraries were evenly pooled and subjected to paired-end sequencing using NextSeq 2000 P1 reagents by Illumina, resulting in the generation of 150-base pair reads. Quality assessment of the reads, species identification, and determination of the multi-locus sequence type (MLST) using the Pasteur scheme (Jolley et al., 2018) (*cpn60, fusA, gltA, pyrG, recA, rplB, rpoB*) were conducted through the Nullarbor bioinformatics pipeline v2 (https://github.com/tseemann/nullarbor). Additionally, the presence of antimicrobial resistance (AMR) genes was investigated using ResFinder (v4.4.2), with criteria set at 90% identity and 60% coverage, at the Center for Genomic Epidemiology (CGE) (https://genomicepidemiology.org/services/), as well as Resistance Gene Identifier (RGI) (v6.0.3) at the Comprehensive Antibiotic Resistance Database (CARD) (v3.2.8) (https://card.mcmaster.ca/home). *AmpC* gene presence was also determined using the PubMLST database (https://pubmlst.org/organisms/acinetobacter-baumannii/. Representative isolates from CR*Ab* carrying *bla*_NDM-1_ underwent supplementary sequencing using the MinION sequencer (Oxford Nanopore Technologies, ONT) (Wang et al., 2021). The resulting ONT reads were combined with the Illumina short reads using the Unicycler hybrid assembly pipeline v0·4·9b to generate complete genomes (Wick et al., 2017). The complete genomes were used to detect the existence of plasmids and other mobile genetic elements (MGEs) (**Supplementary Page 5**).

### Phylogenetic analysis

Phylogenetic relationships among all the isolates based on the core genome were determined by aligning sequences to a complete reference genome of the same sequence type. Core genome variants were identified using Snippy (v.4.6.0), (https://github.com/tseemann/snippy) and recombinant regions from the aligned core genome sequences were masked using Gubbins (v.2.3.4) (Croucher et al., 2015). Non-recombinant core genome single nucleotide polymorphisms (SNPs) were extracted using SNP-sites (Croucher et al., 2015; Page et al., 2016). Genetic clustering of isolates was performed using Fast hierarchical Bayesian analysis of population structure (fastBAPS) (Tonkin-Hill et al., 2019) in RStudio (v4.1.2), and GraphSNP (v1.0) (Permana et al., 2023). The pairwise SNP distance threshold for establishing genetic relatedness was set at ≤19 SNPs, based on findings from our molecular and clinical epidemiological study at the CWMH, which suggested that this threshold was indicative of genetic relatedness among *Acinetobacter baumannii* isolates within the context of an outbreak (Baleivanualala et al., 2023). Maximum-likelihood (ML) phylogeny inference was conducted on the non-recombinant core genome SNPs using FastTree (v2.1.10, double precision [No SSE3]) (Price et al., 2010). The resulting trees were visualized using the Interactive Tree of Life (iTOL) (v6.8.1) (https://itol.embl.de/). To understand the evolution and dissemination of CR*Ab* in Fiji, and to explore connections between environmental and non-duplicate clinical isolates, a phylogenetic analysis was performed by comparing all the CR*Ab* isolates obtained from high-touch surfaces with previously sequenced CR*Ab* obtained from patients at CWMH in 2016/2017 and 2019 - 2021, and LTKH and LBSH in 2020 – 2021 from our recent studies (Baleivanualala et al., 2023, 2024; Zimmerman et al., 2017) (**Supplementary Tables 3**–**5**). In addition, a CR*Ab* isolate obtained from a patient admitted to the adult intensive care unit (ICU) of CWMH that had been collected and stored in 2022 was available for sequencing, so was included; other clinical isolates from the same period were not collected as part of this study and therefore were not available for analysis. In-depth comparisons were also performed within individual hospital settings to enhance the precision of the analysis.

Further detail on bioinformatics analyses is provided in **Supplementary Page 5**.

## Results

Of the 180 samples collected, 94 (52.2%) were from CWMH, 42 (23.3%) from LTKH, and 44 (24.4%) from LBSH (**Supplementary Table 1**). Of the 94 CWMH samples, 70 (74.5%) were from ICU settings, comprising 42 (44.7%) from adult ICU, 11 (11.7%) from maternity ICU (MICU), 10 (10.6%) from pediatric ICU (PICU), and seven (7.4%) from neonatal ICU (NICU). Moreover, 13 (13.8%) were from medical and 11 (11.7%) from surgical inpatient settings. Of the 42 LTKH samples, 21 (50.0%) were from ICU, with 16 (38.1%) from adult ICU and five (11.9%) from PICU. Moreover, 12 (28.6%) were from medical and nine (21.4%) from surgical inpatient settings. The 44 LBSH samples comprised 19 (43.2%) samples from medical settings, 15 (31.1%) from surgical settings, and 10 (22.7%) from ICU, with adult ICU and NICU each contributing five (50%).

### Species identification, antimicrobial susceptibility and carbapenemase screening

Fifty-seven (31.7%) of the 180 samples exhibited growth on MacConkey agar culture plates. Among these, 31 (54.4%) were from CWMH, 17 (29.8%) from LTKH and nine (15.8%) from LBSH. Of the 57 positive samples, 39 (68.4%) were identified as *A. baumannii* complex, 13 (22.8%) as *Escherichia coli*, three (5.3%) as *Klebsiella pneumoniae*, and two (3.5%) as *Pseudomonas aeruginosa*. Sources of these isolates are detailed in **Supplementary Table 1**. Of the 39 A*. baumannii*, ten (25.6%) exhibited resistance to meropenem; all were also resistant to gentamicin, amikacin, ciprofloxacin, and trimethoprim/sulfamethoxazole but were susceptible to colistin. All the CR*Ab* produced a carbapenemase enzyme as determined by AmCIM. In contrast, all *E. coli*, *K. pneumoniae*, and *P. aeruginosa* were susceptible to meropenem. Of the 10 CR*Ab* isolated from various high-touch surfaces (**Table 1**), seven (70.0%) were isolated from CWMH; six (60.0%) from the adult ICU, and one (10%) from the MICU. The remaining three CR*Ab* were isolated from LTKH, with two (20.0%) from a surgical setting and one (10.0%) from a medical inpatient setting. No meropenem resistance was observed among LBSH isolates. When analyzing the proportion of CR*Ab* against the total number of high touch samples collected within the same hospital setting, we found that six (14.3%) CR*Ab* were obtained from the 42 samples from CWMH’s adult ICU and one (9.1%) from the 11 samples from CWMH’s MICU. At LTKH, two (40.0%) of the five and one (25.0%) of the four samples obtained from the surgical and medical inpatient settings respectively yielded a CR*Ab* (**Supplementary Table 1**).

**Table 1:** Phenotypic and molecular characteristics, and source of high-touch isolates of CR*Ab* from CWMH and LTKH (2021 – 2022).

Isolate ID	Date of collection	Hospital	Hospital setting	Sampling Source	Sequence type	Phenotypic antimicrobial resistance profile	Genotypic resistance profile
FJ415_CWE	26/09/2021	CWMH	ICU	Infusion pump screen	2	All‡ except CN	*ampC*_-2_, *bla*_OXA-23_***, *bla*_OXA-66_, *bla*_NDM-1_***, *aadA1*, *aph(3’)-Ia*, *aph(3’)-Ib, aph(6’)-Id*, *armA*, *gyr A (S81L), parC* (S84L, V104I, D105E), *catB8 su1*, *sul2, catB8, tet(B)*, *qacEdelta1*, *ble-MBL*, *mphE*, *ade* (*ACFGHIJKLRS*)^^^, *abaQ*, *abeS*
FJ413CWE	26/09/2021	CWMH	ICU	Emergency trolley top			
FJ412_CWE	26/09/2021	CWMH	ICU	Emergency trolley top	25	All‡ except CN	*ampC*_-25_, *bla*_OXA-23_***, *bla*_OXA-64_, *bla*_PER-7_, *aph(3’)-Ib*, *aph(6’)-Id*, *armA*, *parC* (V104I, D105E), *cmlA5*, *sul1*, *qacEdelt1* *Ade (FJKLRS)*, *abaQ*, *abeS*
FJ414_CWE	26/09/2021	CWMH	ICU	Bed linen			
FJ416_CWE	26/09/2021	CWMH	MICU	Floor (between beds)			
F610CWE	1/12/2022	CWMH	ICU	Workstation tabletop	499	All‡ except CN	*ampC*_-70_, *bla*_OXA-23_***, bla_OXA-95_, *bla*_PER-7,_ *aph(3’)-Ib*, *aph(6’)-Id*, *armA* *parC* (V104I, D105E), *cmlA1*, *cmlA5, su1*, *sul2, tet(B)*, *qacJ* *ade (FGHJKLNR)* ^^^, *abaQ*, *abeS*
FJ611CWE	1/12/2022	CWMH	ICU	Ventilator tube			
FJ615LTE	29/08/2022	LTKH	Surgical	Infusion pump protocol file			
FJ616LTE	29/08/2022	LTKH	Surgical	Patient’s cubicle curtain			
FJ617LTE	29/08/2022	LTKH	Medical	Dressing trolley			

‡Antimicrobials tested: meropenem, piperacillin/tazobactam, amikacin, gentamicin, trimethoprim/sulfamethoxazole, ciprofloxacin, colistin (CN).

***carbapenem resistant gene.

CWMH, Colonial War Memorial Hospital; LTKH, Lautoka Hospital; ICU, intensive care unit; MICU, maternity ICU; CN, colistin; abeS, SMR, small multidrug resistance efflux pump; aph, aminoglycoside phosphotransferase; Arm, aminoglycoside resistance methylase; aac, abaQ, A. baumannii quinolone resistance transporter; Ade, adenine deaminase; ABC, ATP-binding cassette; bla_NDM_, New Delhi metallo-β-lactamase; bla_OXA_, OXA-type β-lactamase; CAT, chloramphenicol acetyltransferase; gyr, DNA gyrase; par, Type IV topoisomerase; qac, quaternary ammonium compounds; mph, macrolide phosphotransferase; MFS, major facilitator superfamily; RND, Resistance Nodulation-Division; sul, sulfonamide resistance gene; tet, tetracycline.

### Phylogenetic analysis

Of the 10 A*. baumannii*, two (20%) belonged to international clone (IC)-2 sequence type (ST2), three (30%) to ST25, and five (50%) to ST499 (**Table 1**).

### Carbapenem resistant *Acinetobacter baumannii* ST2

**Figure 1:**
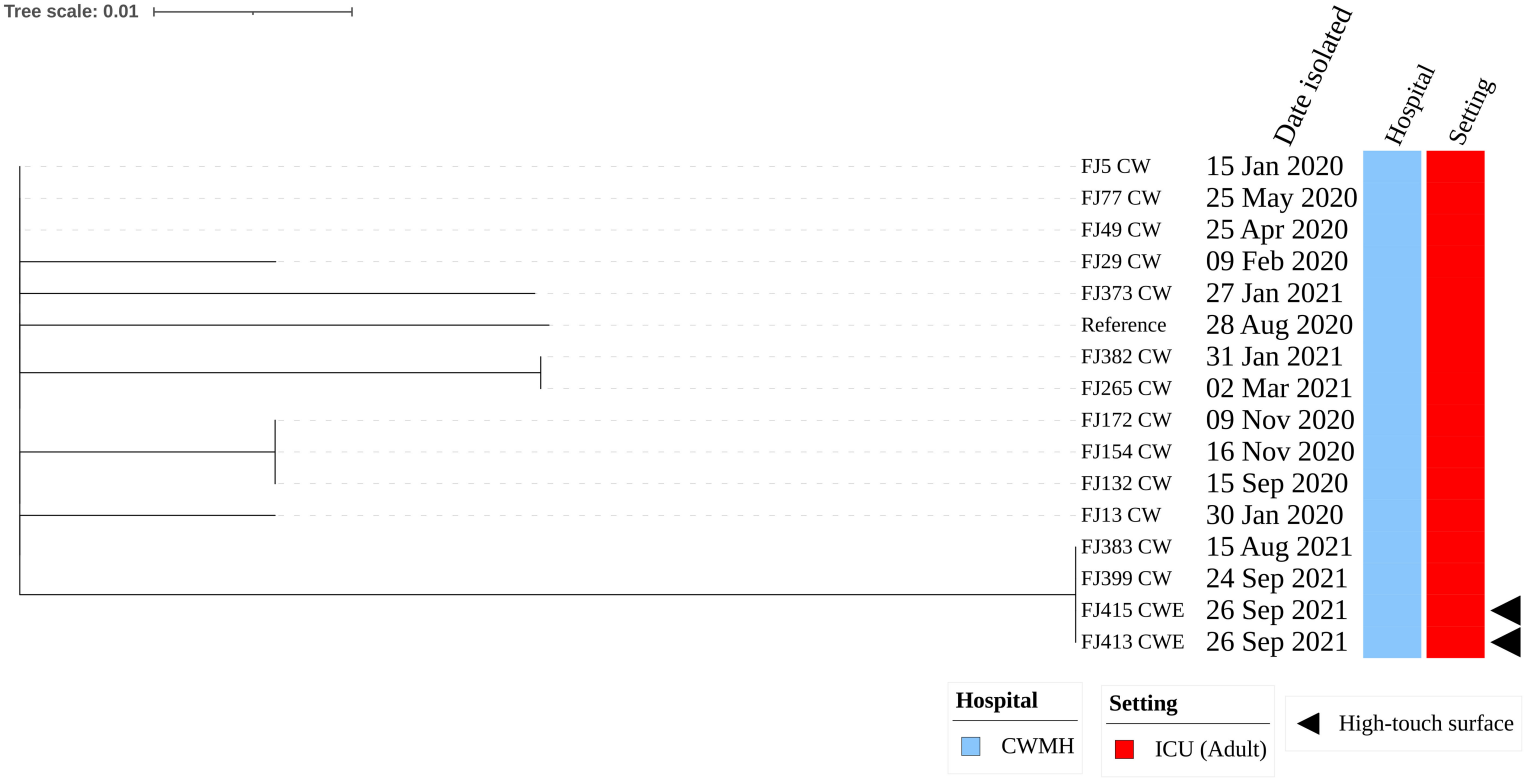
Core genome SNP phylogeny of environmental and clinical carbapenem resistant *Acinetobacter baumannii* ST2 clone 3 from Fiji’s adult ICU CWMH, (2021 - 2022). Core genome SNP phylogeny of carbapenem resistant *A. baumannii* ST2 isolates from adult ICU of CWMH, Fiji (2020 - 21). Phylogenetic tree inferred from core genome SNPs of 15 CR*Ab* ST2 isolates. The core genome was generated by aligning at least 95% of the available sequence data for all isolates. The core SNP density, representing the average number of SNPs per base pair in the core genome, was 13 SNPs across 4080253 base pairs in the reference genome. The reference genome used was a CR*Ab* ST2 (FJ104_CW) isolated from a patient at CWMH in 2020 (Baleivanualala et al., 2024). The tree was rooted using the earliest isolate (reference) from CWMH. Isolation dates, hospital, and setting are annotated on the tree. The scale bar represents the frequency of mutations per site.

The two CR*Ab* ST2 isolates were isolated from one of the infusion pump screens and the emergency trolley within the adult ICU of CWMH. These isolates harbored the insertion sequence element (IS*Aba125*) position upstream of the *bla*_NDM-1_. Moreover, these two environmental CR*Ab* ST2 isolates were genetically identical when compared to a complete genome of CR*Ab* ST2 clone 3 (FJ104_CW), isolated from CWMH in 2020, as the reference (**Supplementary Figure 2A**). When these two CR*Ab* ST2 environmental isolates were compared with the 167 clinical CR*Ab* ST2 isolates collected from various hospitals, including CWMH in 2016/2017 and 2020–2021 (Baleivanualala et al., 2023, 2024; Zimmerman et al., 2017), as well as LTKH and LBSH from 2020 to 2021, they clustered together within the CR*Ab* ST2 clone 3 outbreak strains obtained from CWMH, LTKH, and LBSH in 2020–2021, as outlined in our recent study (Baleivanualala et al., 2023, 2024) (**Supplementary Figure 2B**, **Supplementary Table 3**). Both environmental isolates were closely related to all ST2 clone 3 isolates obtained from patients admitted to CWMH, LTKH, and LBSH in 2020–2021, regardless of hospital setting, with a median (range) difference of 3 (0 - 11) SNPs using a complete genome of CR*Ab* ST2 clone 3 (FJ104_CW), isolated from CWMH in 2020, as the reference (**Supplementary Figure 2C**, **Supplementary Table 3**). Moreover, when the two high-touch isolates were compared to 14 ST2 clone 3 isolates obtained from 14 patients admitted to CWMH’s adult ICU in 2020 - 2021, using the same reference genome, they were closely related with a median (range) difference of 3 (0 - 5) SNPs (**Figure 1**). Of the 14 ST2 clone 3 isolates, two (14%) (FJ383_CW and FJ399_CW) were genetically identical to the two high-touch isolates, with no SNP differences.

### Carbapenem resistant *Acinetobacter baumannii* ST25

**Figure 2:**
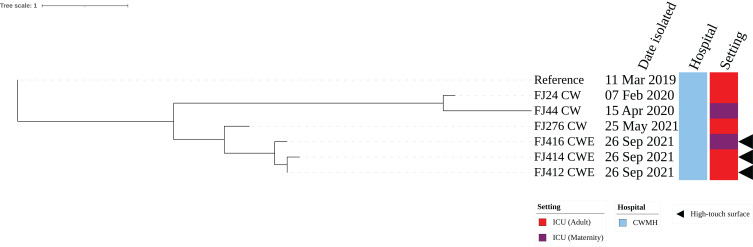
Core genome SNP phylogeny of environmental and clinical carbapenem resistant *A. baumannii* ST25 isolates from adult and maternity ICU of CWMH (2019 – 2021). Phylogenetic tree inferred from core genome SNPs of seven CR*Ab* ST25 isolates. The core genome was generated by aligning at least 94% of the available sequence data for all isolates. The core SNP density, representing the average number of SNPs per base pair in the core genome, was 69 SNPs across 4210148 base pairs in the reference genome. The reference genome used was from CR*Ab* ST25 (FJ14), isolated from an adult ICU patient at the CWMH in 2019 (Baleivanualala et al., 2023). The tree was rooted using the earliest isolate (reference genome, FJ14). Isolation dates and locations are annotated on the tree. The scale bar represents the frequency of mutations per site.

The three CR*Ab* ST25 isolates were all obtained from CWMH, with two (66.7%) originating from the adult ICU, one from an emergency trolley and the other from bed linen used by a patient with CR*Ab*. The third isolate was obtained from the floor between patients’ beds in the MICU. All the three environmental isolates were closely related with a median (range) difference of 0.5 (0 – 2) SNPs using a CR*Ab* ST25 (FJ14) isolated from CWMH in 2019 as the reference (**Supplementary Figure 3A**). When comparing the three ST25 isolates to the 15 CR*Ab* ST25 isolated from patients admitted to the CWMH in 2019–2021 and LBSH in 2020, using the complete genome of FJ14 as the reference, they all clustered together with the CR*Ab* ST25 clone 2 strains outlined in our recent study (Baleivanualala et al., 2023) (**Supplementary Figure 3B**, **Supplementary Table 4**). The three environmental isolates were compared to two ST25 (FJ24 CW and FJ276 CW) isolated from two patients admitted to CWMH’s adult ICU setting and to one (FJ44 CW) isolated from CWMH’s maternity ICU in 2020 - 2021, using the same reference genome (**Figure 2**). The two CR*Ab* ST25 isolates (FJ412CWE, FJ414CWE) from the adult ICU environment differed by 31 and 32 SNPs, respectively, from the CR*Ab* ST25 isolate (FJ24CW) obtained from a patient in the same ICU setting in 2020 (**Figure 2**). However, they (FJ412CWE, FJ414CWE) were closely related to the ST25 isolate (FJ276CW) collected from a patient admitted to the same ICU setting 116 days prior to the environmental sampling date by 7 and 8 SNPs respectively (**Figure 2**).The isolate (FJ416CWE) obtained from the floor between patients’ beds in the MICU differed by 37 SNPs from an isolate (FJ44CW) collected from a patient admitted to the same maternity ICU in 2020 but was closely related (7 SNPs) to the isolate from the adult ICU from 2021 (FJ276CW).

### Carbapenem resistant *Acinetobacter baumannii* ST499

**Figure 3:**
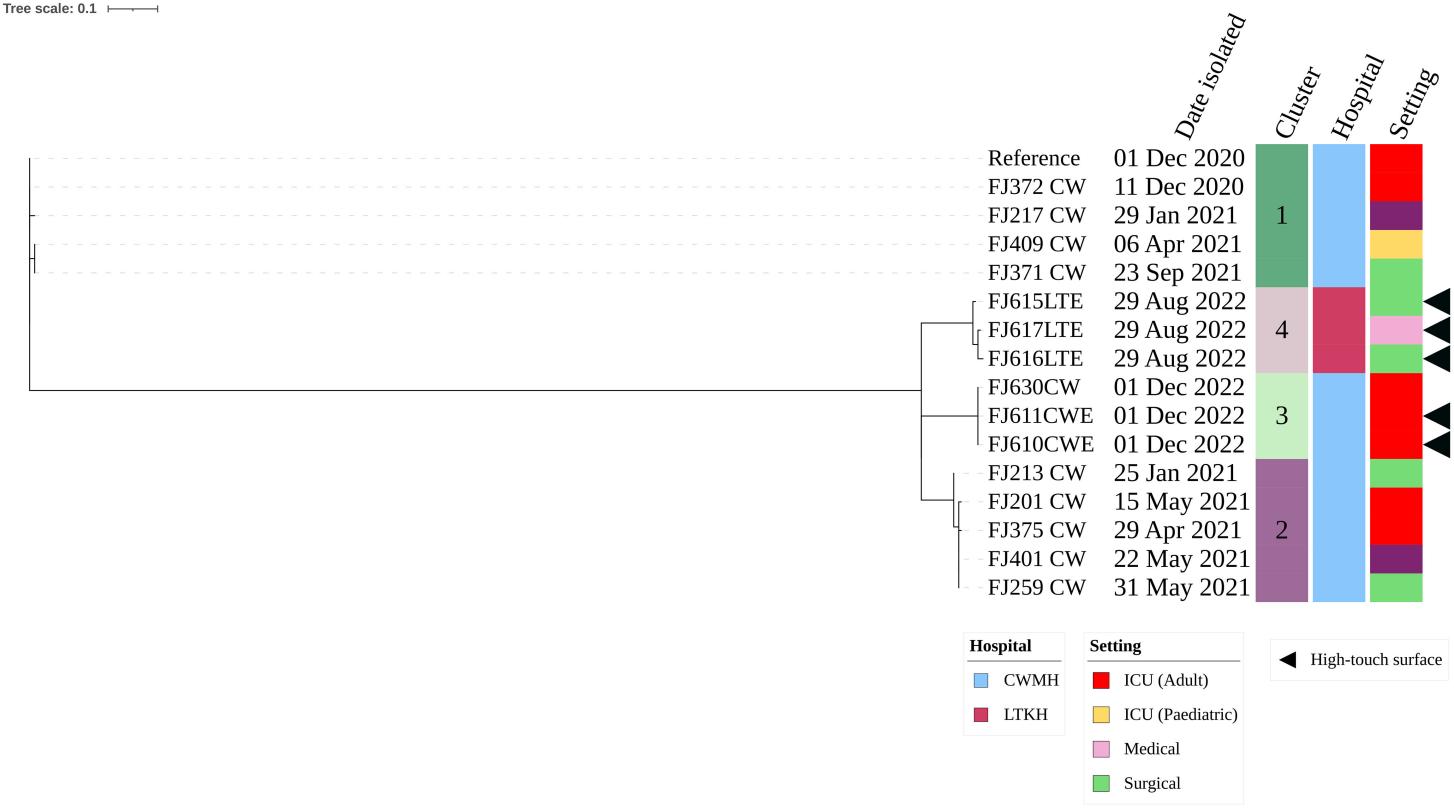
Core genome SNP phylogeny of environmental and clinical carbapenem resistant *A. baumannii* ST499 isolates from CWMH (2020 - 2021). Phylogenetic tree inferred from core genome SNPs of 15 CR*Ab* ST499 isolates. The core genome was generated by aligning at least 94% of the available sequence data for all isolates. The core SNP density, representing the average number of SNPs per base pair in the core genome, was 2064 SNPs across 4274662 base pairs in the reference genome. The reference genome used was CR*Ab* ST499 (FJ275_CW), isolated from a patient in CWMH in 2020. The tree was rooted using the earliest isolate (reference). Isolation dates, locations, collection site, are annotated on the tree. The scale bar on the tree represents the frequency of mutations per site, providing a measure of genetic distance between isolates.

Of the five CR*Ab* ST499, three (60.0%) were isolated from LTKH and two (40.0%) from CWMH. Within LTKH, two of (66.7%) the three ST499 strains were isolated from various locations within the surgical ward, specifically from a cubicle curtain near a patient’s bed, an infusion protocol file, and the tray shelf of the dressing trolley. The third ST499 strain was isolated from the dressing trolley used in medical ward. The two CR*Ab* ST499 isolates from CWMH were found on a central workstation tabletop and a ventilator tube used by a patient in the adult ICU. Our phylogenetic analysis showed the presence of two distinct clones: one consisting of two genetically identical isolates from the adult ICU of CWMH, and the second comprising three closely related isolates from LTKH with a median (range) difference of 1.5 (0 – 5) SNPs (**Supplementary Figure 4**). Upon comparing the five CR*Ab* ST499 isolates with 15 CR*Ab* ST499 obtained from patients admitted in CWMH in 2020 – 2021,^16^ the analysis revealed four distinct clones, two of which included environmental isolates (**Figure 3**, **Supplementary Table 5**). Notably, one patient isolate (FJ630_CW) was genetically identical to the two CWMH environmental isolates (FJ610CWE and FJ611CWE) and formed clone 3 (**Figure 3**); isolate FJ630_CW was from the same patient in CWMH’s ICU during the collection period whose ventilator tubing yielded one of the environmental isolates (FJ611CWE).

## Discussion

Our study sheds light on the contamination with CR*Ab* of high-touch environment and equipment surfaces within intensive care, medical, and surgical healthcare settings in Fiji hospitals. The identification of multiple CR*Ab* clones, including ST2, ST25, and ST499, exhibiting resistance to multiple classes of antimicrobials, including carbapenems, signifies a complex and challenging scenario for infection prevention and control. The persistence of these resistant strains in ICU, medical, and surgical inpatient settings, indicates that these hospital environments may serve as sources for CR*Ab*, although contamination of the environment by patients who have acquired CR*Ab* from other sources cannot be ruled out. This is a critical concern, as these clones are not only present on high touch surfaces but are also phylogenetically linked to clinical CR*Ab* isolates from previously reported nosocomial outbreaks, occurring in Fiji’s hospitals since 2019 (Zimmerman et al., 2017); Baleivanualala et al., 2023. The phylogenetic links between environmental CR*Ab* and those causing clinical infections highlight a potential association between environmental contamination and subsequent infections, raising questions about the efficacy of current infection prevention and control measures in Fiji.

High-touch surfaces and equipment in healthcare facilities, such as door handles, bed rails, light switches, sinks, bed linens, curtains, infusion pumps, and ventilators, are recognized as hotspots for bacterial transmission (O’Neil et al., 2017; Ng et al., 2018). Of note, we detected CR*Ab* ST2, ST25, and ST499 on various high-touch surfaces within the adult and maternity ICU, as well as medical and surgical inpatient settings of CWMH and LTKH. The presence of genetically identical CR*Ab* ST2 on the infusion pump and emergency trolley, and phylogenetic association with an isolate from a patient discharged four weeks prior to environmental sampling, highlights its potential persistence in the hospital. These CR*Ab* ST2 isolates harbored the insertion sequence element (IS*Aba125*) positioned upstream of the *bla*_NDM-1_ gene, contributing to a high level of carbapenem resistance, consistent with findings from CR*Ab* ST2 clone 3 identified in our recent study (Baleivanualala et al., 2024). However, not all CR*Ab* colonization or infection events were detected, suggesting some instances may have been overlooked. This persistence is further supported by its close phylogenetic relationship to isolates obtained from patients in CWMH, LTKH, and LBSH since 2020. The detection of closely related CR*Ab* ST25 isolates on the floor of MICU and in the adult ICU on bed linen used by a patient and an emergency trolley top raises concerns about transmission between units within the facility. Although the MICU and ICU do not share medical staff, they have common non-medical staff, such as cleaners. This shared staff interaction could potentially explain why these two closely related strains were discovered in these wards although acquisition from other sources cannot be ruled out (Mboowa et al., 2021). This emphasizes the importance of utilizing effective cleaning and disinfection practices for floors, along with ensuring regular linen changes and thorough disinfection protocols, to effectively mitigate the spread of this persistent pathogen. The phylogenetic linkage of ST25 isolates from the high touch surfaces to those obtained from patients since 2020, including those admitted more than three months before sampling, raises concerns about ongoing transmission dynamics and the potential for recurrent outbreaks. The identification of genetically related CR*Ab* ST499 on both a cubicle curtain and on infusion pump protocol file at the LTKH suggests a potential common source or transfer of ST499 between these surfaces suggesting that HCWs may serve as intermediaries, facilitating the transmission of CR*Ab* between these fomites. Given that the protocol file, which is typically stored in a cupboard and accessed on an as-needed basis, also harbored CR*Ab*, it further supports the notion of transmission via HCWs’ contaminated hands or gloves. To support this, further investigation involving the swabbing of HCWs’ hands is necessary. Considering CR*Ab*’s ability to persist for at least four months on dry surfaces (Wendt et al., 1997; Katzenberger et al., 2021), establishing a systematic, scheduled protocol for the replacement of cubicle curtains and ensuring the sterile storage of unused curtains become essential actions. Moreover, enforcing stringent hand hygiene protocols is crucial. CR*Ab*’s association with ventilator-associated pneumonia in ICU settings is well-established (Nhu et al., 2014; Pogue et al., 2022). The presence of genetically identical CR*Ab* ST499 strains on the workstation tabletop, ventilator, and a patient suggests a direct link and potential transmission pathway involving medical equipment, the hospital environment, and HCWs who may unintentionally spread the pathogen via contaminated hands or gloves. The detection of CR*Ab* ST2, ST25, and ST499 on emergency and dressing trolleys in hospital settings, where trolleys are shared across various patient care activities within the same hospital setting, raises concerns about their potential to spread these resistant strains. Although all three hospitals adhere to standard operating procedures and guidelines for disinfection, there remains a risk of AMR contamination in the environment. This risk is heightened by the possibility of suboptimal disinfection practices. Moreover, the continued reuse of single-use equipment further exacerbates the risk of spreading resistant strains, as these items may not be adequately disinfected between uses. However, our analysis did not extend to isolates from patient or HCW hand swabs, thus no directionality of transmission via shared trolley use or hand contact is evident. This issue gains significance in light of our recent findings indicating a surge in hospital-acquired infections with CR*Ab*, especially those related to wound infections.

Interestingly, all the high touch surface CR*Ab* strains encoded the quaternary ammonium compound resistance gene (*qacEdelta1*, *qacJ*), which confers resistance to widely used hospital disinfectants such as benzalkonium chloride, benzethonium, and chlorhexidine and other QAC based disinfectants. The existence of this *qac* gene raises concerns about the efficacy of routine disinfection practices employing these common disinfectants in healthcare settings (Tandukar et al., 2013; Elkhatib et al., 2019). This issue is of great concern, considering the recent findings on the ongoing challenges with hand hygiene compliance of HCWs in the three hospitals, which increases the risks of spreading these resistant strains (Zimmerman et al., 2017; Zimmerman, 2018). Moreover, this situation is further exacerbated by the inconsistencies of supplies of effective alcohol-based handrub (Loftus et al., 2020).

While our study revealed the presence of multiple strains of CR*Ab* in the hospital environment in Fiji, it had limitations. First, the investigation into transmission pathways was not exhaustive. We did not investigate other transmission routes, such as via healthcare worker hands, patient-to-patient transmission, and colonization of plumbing systems. Since we did not screen patients for possible CR*Ab* colonization, we were unable to observe complete chains of transmission. Second, despite records indicating the presence of CR*Ab* in hospital settings during the environmental study period, isolates from patients admitted to respective hospitals setting during the environmental study period were not part of our study. Therefore, we do not know the prevalence of patients colonized and infected with CR*Ab* at the time of the study and cannot track transmission between patients. Third, the uneven distribution of samples between hospitals and the use of professional judgment for estimating the sampling area, which may introduce variability and bias. Moreover, the lack of comparability between sampling locations limits our ability to conclusively identify specific sources and their role in transmission. Future research should focus on standardized comparisons across different sites to address these limitations. Fourth, the absence of post-cleaning swabs hindered the assessment of the effectiveness of disinfection procedures and the possibility of finding the sources of these CR*Ab* strains. Fifth, we did not specifically assess the IPC protocols in this study, but future research could explore the impact of these differences on environmental contamination and transmission dynamics. These limitations underscore the importance of addressing these aspects in a future study for a more comprehensive understanding of AMR dynamics in healthcare settings in Fiji.

Our study has revealed the widespread presence of CR*Ab* on high-touch surfaces throughout ICU, medical, and surgical settings in Fiji’s healthcare facilities, posing a risk of hospital-acquired infections and potential transmission beyond the hospital environment. While the directionality of CR*Ab* transmission remains unclear, the phylogenetic linkages observed between CR*Ab* strains from patients (Zimmerman et al., 2017; Baleivanualala et al., 2023) and high-touch surfaces suggests plausible transmission pathways within healthcare settings. The detection of CR*Ab* on diverse surfaces raises concerns about possible hand and glove contamination, emphasizing the risk of transmission. Implementing surveillance by AMR screening of new admissions and regular sampling of admitted patients could provide a more robust design to elucidate the direction of transmission between patients and the environment. This approach would enhance our understanding of transmission dynamics and inform targeted infection prevention strategies. The existence of potential sources for these resistant strains, encompassing emergency trolleys, dressing trolleys, workstations, cubicle curtains, bed linens, floors, and various medical equipment, underscores the urgent need for rigorous infection prevention and control measures. To counter this threat, consistent adherence to environmental sanitation and disinfection protocols, especially for high-touch surfaces, is essential. This involves using effective sanitation and disinfectant agents with careful attention to proper application and sufficient contact time (Mohapatra, 2017). Strict adherence to hand hygiene protocols, incorporating proper handwashing techniques and effective disinfectants, is crucial for HCWs. Establishing routine and thorough disinfection procedures for hospital linens, including bed sheets and curtains, is vital. This should align with established guidelines for laundering practices, utilizing suitable water temperatures, and antimicrobial detergents (https://www.cdc.gov/hai/prevent/resource-limited/laundry.html) (Bockmühl et al., 2019). In addition to these measures, routine environmental screening for AMR pathogens is crucial. It enables early detection of resistant strains on surfaces and equipment, prompting targeted interventions. Identifying AMR reservoirs helps refine infection control strategies, improve cleaning protocols, and reduce transmission risks to patients and staff. Continuous training initiatives and public awareness campaigns should emphasize the critical importance of hand hygiene among HCWs and visitors coming to the hospital. Recognizing the imperative role of hospital management in providing consistent supply of appropriate consumables and other products for infection prevention and control practice and adequate staffing is crucial for effectively addressing this issue. Moreover, management’s commitment to leading infection prevention and control efforts, promoting its significance, and enforcing stringent protocols within their hospitals is vital for mitigating these issues. A collaborative effort among HCWs, hospital management, and the wider community is required to safeguard public health in Fiji, underscoring the collective responsibility in preventing the spread of AMR pathogens and maintaining a safe healthcare environment.

## Data Availability

The datasets presented in this study can be found in online repositories. The names of the repository/repositories and accession number(s) can be found below: https://www.ncbi.nlm.nih.gov/, PRJNA1102903.
