# Supplementary material for: Environmental contamination with carbapenem resistant Acinetobacter baumannii in healthcare settings in Fiji: a potential source of infection
Source: Front Cell Infect Microbiol. 2024 Sep 23;14:1429443. doi: 10.3389/fcimb.2024.1429443 (PMC11456574; doi:10.3389/fcimb.2024.1429443)
Supplement: Supplementary file 1 [file Table1.docx]

*Supplementary material*

Environment contamination with carbapenem resistant *Acinetobacter baumannii* in healthcare settings in Fiji: a potential source of infection

Sakiusa C. Baleivanualala^1,2,3^, Silivia Matanitobua^4^, Yvette Samisoni^5^, Vika Soqo^4^, Shayal Smita^4^, Josese Mailulu^6^, Ilisapeci Nabose^4^, Alvina Lata^4^, Christina Shayam^4^, Radhika Sharma^4^, Donald Wilson^2^, John A. Crump^7,8^, *James E. Ussher^1,3,9^

1. Department of Microbiology and Immunology, School of Biomedical Sciences, University of Otago, Dunedin 9054, New Zealand.

2. College of Medicine, Nursing and Health Science, Fiji National University, Suva, Fiji.

3. Maurice Wilkins Centre for Molecular Biodiscovery, University of Auckland, Auckland 92019, New Zealand

4. Ministry of Health, Fiji

5. Aspen Medical, Lautoka, Fiji

6. Pacific Laboratory Specialist, Suva, Fiji

7. Centre for International Health, Division of Health Sciences, University of Otago, Dunedin, New Zealand.

8. Otago Global Health Institute, University of Otago, Dunedin 9054, New Zealand

9. Awanui Labs, Dunedin Hospital, Dunedin, New Zealand.

*** Correspondence:**Corresponding Author
james.ussher@otago.ac.nz

**Keywords:** Carbapenems, antimicrobial resistance, *Acinetobacter baumannii*, Fiji, hospital environment, hospital-acquired infection

**Study design**

We conducted a molecular epidemiological analysis focusing on carbapenem-resistant *Acinetobacter baumannii* (CR*Ab*) isolates. The samples were obtained from high-touch environmental surfaces (environment and medical equipment) in various hospital settings, including the intensive care unit (adult, maternity, paediatrics, and neonatal), as well as other medical and surgical inpatient settings. The study spanned across three major hospitals in Fiji: Colonial War Memorial Hospital (CWMH), Lautoka Hospital (LTKH), and Labasa Hospital (LBSH) **(Supplementary Figure 1)**. Colonial War Memorial Hospital (CWMH) in Suva is the largest with 523 beds, offering advanced and specialized care while serving as a referral centre for LTKH and LBSH. LTKH, with 200 beds, caters to the Western division, and LBSH, with 150 beds, serves the Northern division of Fiji. These hospitals play vital roles in delivering healthcare services, meeting regional needs, and providing training for healthcare professionals in Fiji. Additionally, these hospitals serve as microbiology laboratory hubs, extending testing services to smaller peripheral centres nationwide.

**Supplementary Figure 1. Map of Fiji.**


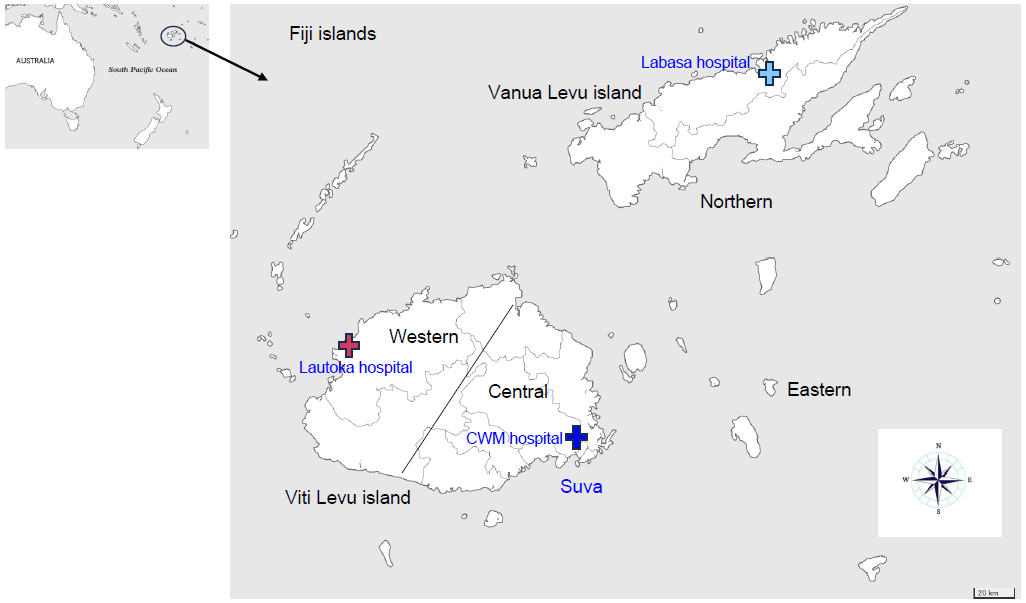


**Reprinted from The Lancet Regional Health - Western Pacific, 47, Baleivanualala *et al*, Molecular and clinical epidemiology of carbapenem resistant *Acinetobacter baumannii*, *Pseudomonas aeruginosa* and Enterobacterales in Fiji: a multicentre prospective observational study, 101095, Copyright (2024), with permission from Elsevier.**

**Sample acquisition**

Total of 180 samples were collected from high-touch environmental and medical equipment surfaces from intensive care units (adult, maternity, paediatrics, and neonatal) as well as medical and surgical inpatient settings (**Supplementary Table 1**). Samples were collected on two occasions (26 September 2021 and 1 December 2022) at Colonial War Memorial Hospital (CWMH) and on a single occasion each (on 29 August and 5 November 2022, respectively) from Lautoka Hospital (LTKH) and Labasa Hospital (LBSH). To standardize the process of environmental sampling for the identification of antimicrobial-resistant (AMR) gram-negative pathogens, we developed a standard operating procedure. The swab was dipped into a vial of tryptic soy broth to moisten it before being rubbed onto the specified location at a 30° angle (Kwan K, Cooper M, La Duc MT, Vaishampayan P, Stam C, Benardini JN, Scalzi G, Moissl-Eichinger C, 2011). The swabbing was performed slowly and thoroughly over approximately 50 cm^2^, utilizing a combination of horizontal, vertical, and diagonal sampling patterns. Swab rotation was implemented during the process (Kwan K, Cooper M, La Duc MT, Vaishampayan P, Stam C, Benardini JN, Scalzi G, Moissl-Eichinger C, 2011). Following environmental swabbing, the swab was carefully transferred into TSB. The remaining segment of the swab stick was broken off, leaving only the swab immersed within the TSB. Subsequently, the broth was incubated aerobically for 24 hours at 37°C. Following incubation, a drop of the tryptic soy broth was inoculated onto MacConkey agar, and the agar was incubated aerobically for 24 hours at 37°C. Colonies were then collected using a charcoal Amies Stuart swab and sent to the University of Otago for further analysis. All swabs received were sub-cultured onto the blood agar culture media and incubated aerobically for 24 hours at 37°C. Isolates were identified using matrix-assisted laser desorption ionization-time of flight (MALDI-TOF) mass spectrometry (Biotyper; Bruker Daltonics, Billerica, MA, USA). Antimicrobial susceptibility testing (AST) was conducted using the EUCAST method, (<https://www.eucast.org/>) and carbapenemase activity detection was performed using the adjusted modified carbapenem inactivation method (mCIM) (Nguyen Vu, Thao Byun, Jung Hyun D’Souza, Roshan Pinto, Naina Adren Nguyen, Le Phuong Yong, Dongeun Chong, 2020). These analyses took place at Awanui Labs (formerly Southern Community Laboratories), Dunedin, New Zealand. For *Acinetobacter baumannii*, susceptibility to meropenem, gentamicin, amikacin, trimethoprim/sulfamethoxazole, ciprofloxacin, and colistin was assessed. For *Pseudomonas aeruginosa*, susceptibility to piperacillin/tazobactam, ceftazidime, cefepime, meropenem, gentamicin, ciprofloxacin, and colistin was evaluated. For the Enterobacterales (*Escherichia coli* and *Klebsiella pneumoniae*), susceptibility to ampicillin, piperacillin/tazobactam, ceftriaxone, meropenem, gentamicin, amikacin, trimethoprim/sulfamethoxazole, ciprofloxacin, and colistin was assessed.

**Standard operating procedure for environment swabbing**

| **Hospital environmental swabbing SOP**  **1.0 Aim**  To guide infection control officer and other health care worker in performing environment sampling for identification of the source of AMR pathogens  **2.0 Principle**  Detection of sources of pathogens is important in the control and prevention of the transmission of AMR pathogens. It also helps in targeting intervention. Using correct sampling procedure will enhance detection of targeted pathogens.  **3.0 Responsibility:** IPC officer/ HCW  **4.0 Sample Requirement:** Environment swabbed samples  **5.0 Equipment/Reagents/Chemical**  5.1 3-5ml sterile Tryptic soy broth (TSB)  5.2.1 Sterile swab, Charcoal Amies transport swab / non charcoal Copan transport swab  **6.0 Procedure**  6.1 Using aseptic technique, dip the sterile swab in the TSB vials provided by Microbiology lab to moisten the swab and then swab the specified location. Press out the excess solution against the interior wall of the vial with a rotating motion.  6.2 Hold the swab handle to make a 30° angle contact with the surface. Aseptically rub the surface slowly and thoroughly over approximately 50 cm^2^ of the surface three times, reversing direction between strokes or S-strokes. Direction of swabbing should be from horizontal to vertical and then to a diagonal sampling pattern. Ensure you rotate the swab during swabbing.  6.3 Return swab into the swab using aseptic technique. Transport swab immediately to the Microbiology Lab under room temperature.  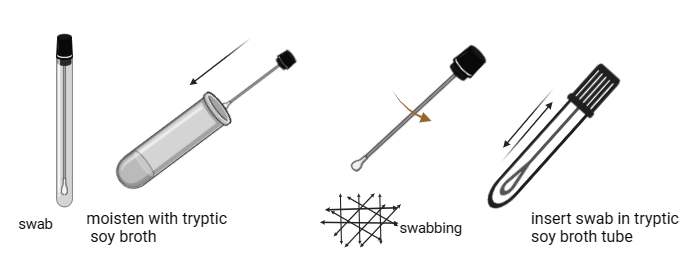  **7.0 Safety Precautions:**  Personnel performing the test should wear standard PPE |
| --- |

**Supplementary Table 1. Refer to excel for table presenting all the high touch environmental and equipment surface data.**

**Supplementary Table 2. Antimicrobial susceptibility testing (EUCAST v11·0 standard guideline, 2021)**

| **Organism** | **Antimicrobial disk** | **Disk content (ug)** | **Zone diameter** | |
| --- | --- | --- | --- | --- |
|  |  |  | **S**  **(mm)** | **R (mm)** |
| *P. aeruginosa* | Ceftazidime  Piperacillin/tazobactam  Ciprofloxacin  Amikacin  Meropenem | 10  30/6  5  30  10 | ≥50  ≥50  ≥50  ≥15  ≥20 | <17  <18  <26  <15  <14 |
| *A. baumannii* | Gentamicin  Amikacin  Trimethoprim/sulfamethoxazole  Ciprofloxacin  Meropenem | 10  30  1·25/23·75  5  10 | ≥17  ≥19  ≥14  ≥50  ≥21 | <17  <19  <11  <21  <15 |
| Enterobacterales | Ampicillin  Piperacillin/tazobactam  Ceftriaxone  Gentamicin  Amikacin  Trimethoprim/sulfamethoxazole  Ciprofloxacin  Meropenem | 10  30/6  30  10  30  1·25/23·75  5  10 | ≥14  ≥20  ≥25  ≥17  ≥18  ≥14  ≥25  ≥22 | <14  <20  <22  <17  <18  <11  <22  <16 |

**The table presents the antimicrobial agents utilized to assess susceptibility in *A. baumannii*, *P. aeruginosa*, and Enterobacterales (including *E. coli* and *K. pneumoniae*). It also includes the EUCAST-established breakpoints for disk-based antimicrobial susceptibility testing.**

**DNA extraction and whole genome sequencing**

Bacterial isolates were plated onto LB agar (Lennox L agar) and incubated overnight at 37^◦^C. Genomic DNA was extracted from overnight cultures using the NucleoSpin® Tissue kit (MACHEREY-NAGEL, Düren, Germany) following the manufacturer’s instructions. The quality and quantity of DNA was determined by Qubit 4·0 Fluorometer (Invitrogen, Thermo Fisher Scientific, Singapore) and Nanodrop (ThermoFisher Scientific, MA, USA). Sample integrity and purity were also assessed by agarose gel electrophoresis (concentration of agarose gel: 1%; voltage: 150 V; electrophoresis time: 40 min). DNA samples were submitted to the Otago Genomics Facility at the University of Otago in Dunedin, New Zealand, for the purpose of library construction and sequencing using the Illumina platform. Representative isolates from CR*Ab* carrying *bla*_NDM-1_ underwent supplementary sequencing using the MinION sequencer (Oxford Nanopore Technologies, ONT). The resulting ONT reads were combined with the BGI short reads using the Unicycler hybrid assembly pipeline v0·4·9b to generate complete genomes (Wick et al., 2017). The complete genomes were used to detect the existence of plasmids and other mobile genetic elements (MGEs).

Representative isolates from CR*Ab* carrying *bla*_NDM-1_ underwent supplementary sequencing using the MinION sequencer (Oxford Nanopore Technologies, ONT) (Wang et al., 2021). DNA libraries were prepared using the ONT Rapid Barcoding Sequencing kit (SQK-RBK110·96) and sequenced using R9·4 (FLO-MIN106) flow cells (Oxford Nanopore Technologies) The sequencing run was conducted using MinKNOW software (v22·12·7). The resulting ONT reads were combined with the BGI short reads using the Unicycler hybrid assembly pipeline v0·4·9b to generate complete genomes (Wick et al., 2017). The complete genomes were used to detect the existence of plasmids and other mobile genetic elements (MGEs).

**Bioinformatics analysis**

The sequence reads' quality, species identification, multilocus sequence type (ST), and phylogenetic relationships of the isolates were assessed using the Nullarbor bioinformatics pipeline version 2.0 (<https://github.com/tseemann/nullarbor>). Initially, raw sequence reads underwent quality and adapter contamination checks with Trimmomatic (v.0.39) to eliminate low-quality bases and adapters. Cleaned reads were then mapped against a known genome database using centrifuge (v1.0.3-beta) for taxonomic labels or species identification. Subsequently, the cleaned reads were assembled into contigs using SPAdes (v3.14.0) and annotated with Prokka (v1.14.0) to predict coding regions and gene functional annotation (<https://github.com/tseemann/prokka>). In silico multilocus sequence typing (MLST) was performed using mlst v2.8.4 and the pubMLST sequence type (ST) database based on specific housekeeping genes for *A. baumannii* (*cpn60, fusA, gltA, pyrG, recA, rplB, rpoB*) (Larsen et al., 2012). AMR genes were detected using ABRicate (v0.9.8) (<https://github.com/tseemann/abricate>) and databases such as ResFinder (<https://genomicepidemiology.org/services/>) and the Comprehensive Antibiotic Resistance Database (CARD) (<https://card.mcmaster.ca/home>) (Alcock et al., 2020). The presence of *ampC* alleles was determined using the PubMLST database of *A. baumannii* (<https://pubmlst.org/organisms/acinetobacter-baumannii/>). For phylogenetic analysis, reads were mapped to a complete reference genome with Snippy (v.4.6.0) (<https://github.com/tseemann/snippy>) to identify the core genome and single nucleotide polymorphisms (SNPs). Recombinant regions were then filtered and removed with Gubbins (v2.3.4) (Croucher *et al.*, 2015) from the whole genome SNP alignment. Core genome SNPs were extracted using SNP-sites, (Page et al., 2016) and SNP distances between genomes were calculated using pairwise SNP distance matrix (<https://github.com/tseemann/snp-dists>). A maximum-likelihood phylogenetic tree was constructed from core SNPs with FastTree (v2.1.10, double precision [No SSE3]) and the resulting trees were visualized using the Interactive Tree of Life (iTOL) (v6.8.1) (<https://itol.embl.de/>). Analyses included only samples with at least 50x depth of coverage and 90% genome coverage**.**

**Supplementary Tables 3**. **Refer to excel for metadata for clinical CR*Ab* ST2 isolates collected from CWMH in 2016/2017 and 2019 to 2021, as well as LTKH and LBSH from 2020 to 2021.**

**Supplementary Table 4. Refer to excel for metadata for clinical CR*Ab* ST25 isolates collected from CWMH in 2019to 2021, as well as LBSH from 2020 to 2021.**

**Supplementary Table 5. Refer to excel for metadata for clinical CR*Ab* ST499 isolates collected from CWMH in 2020 to 2021.**

**Supplementary Figure 2. Core genome SNP phylogeny of environmental and clinical carbapenem resistant *Acinetobacter baumannii* ST2 from Fiji’s CWMH, (2016/2017, 2019, 2020 – 2022).**

**a.**


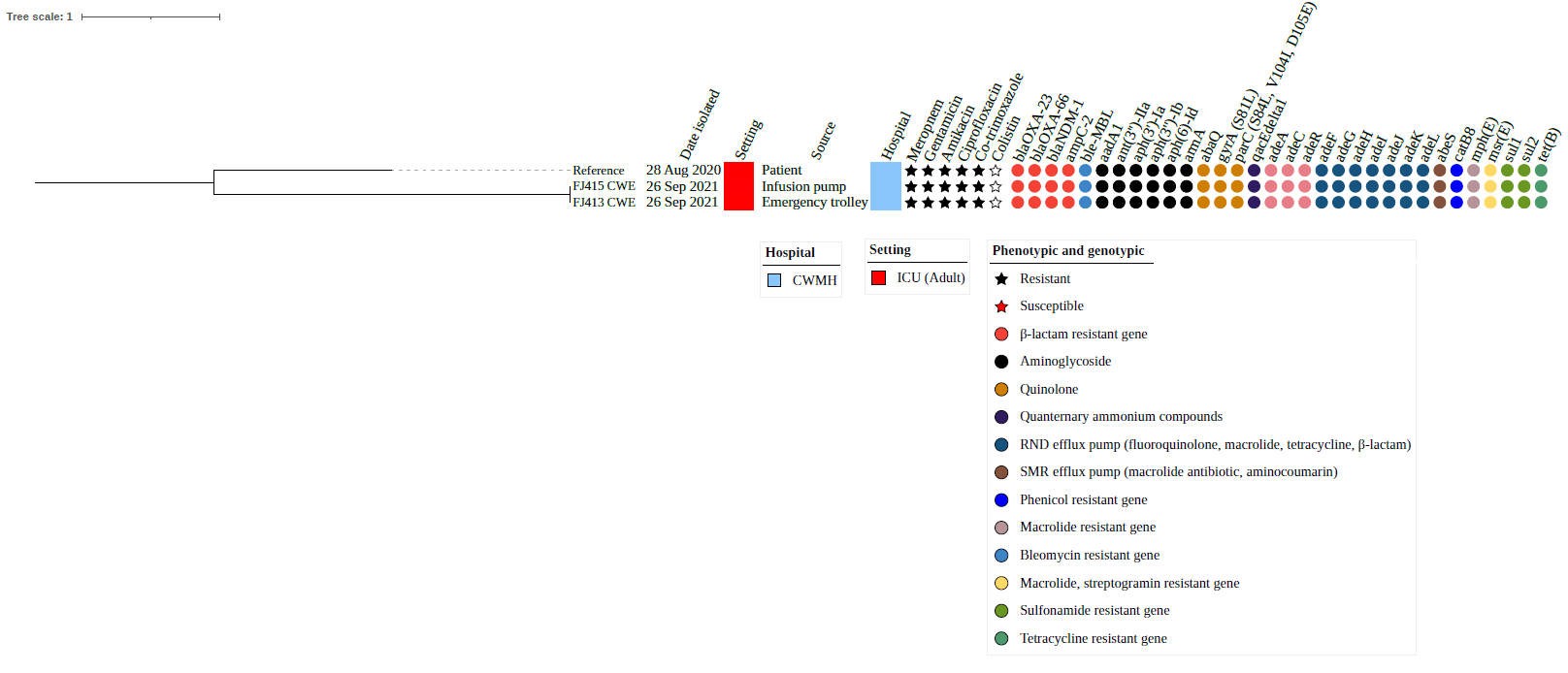


**b.**


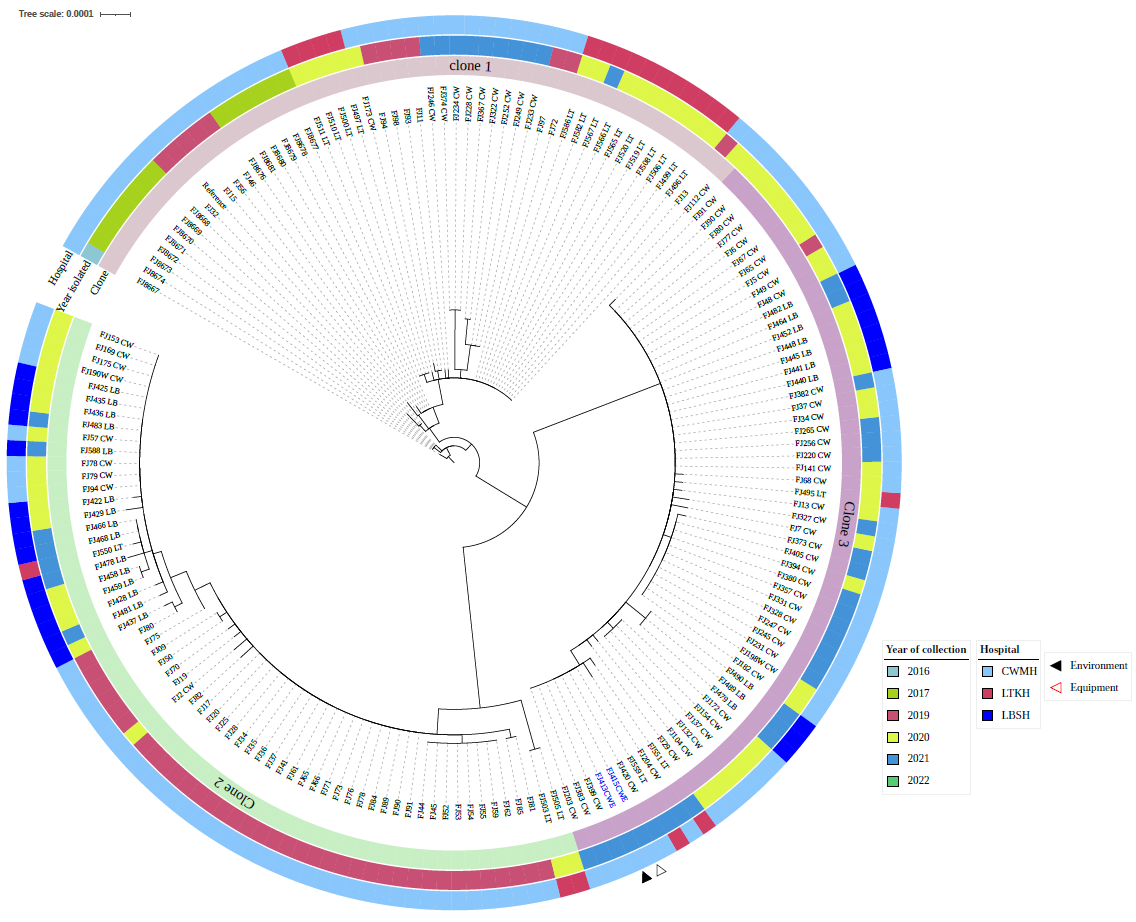


**c.**


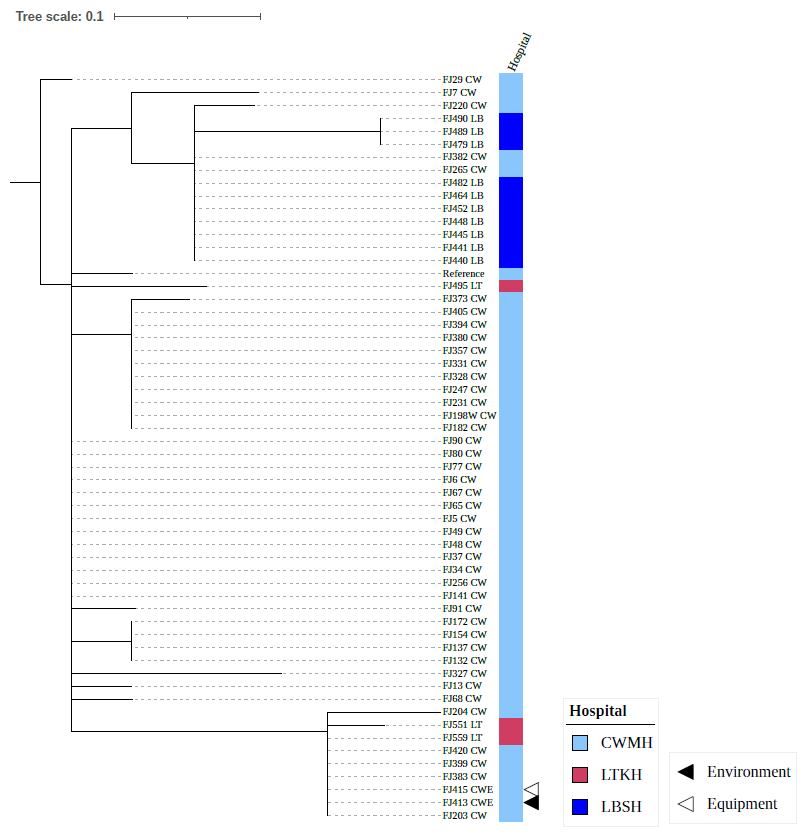


1. **Core genome SNP phylogeny of environmental carbapenem resistant *Acinetobacter baumannii* ST2 from Fiji’s CWMH, (2021). Phylogenetic tree inferred from core genome SNPs of 3 CR*Ab* ST2 isolates. The core genome was generated by aligning at least 95% of the available sequence data for all isolates. The reference genome used was a CR*Ab* ST2 (FJ104_CW) isolated from a patient at CWMH’s adult ICU in 2020** (Baleivanualala et al., 2024)**. Both environmental isolates were genetically identical with 0 SNPs. The tree was rooted using the earliest isolate (reference genome, FJ104_CW). Isolation dates, locations, collection site, and AMR phenotypic and genotypic profiles are annotated on the tree. The scale bar represents the frequency of mutations per site.**
2. **Core genome SNP phylogeny of environmental and clinical carbapenem resistant *A. baumannii* ST2 isolates from CWMH (2016/2017, 2019 – 2022), LTKH, and LBSH (2020 - 2022). Phylogenetic tree inferred from core genome SNPs of 167 CR*Ab* ST2 isolates obtained from patients and high touch surface and equipment. The core genome was generated by aligning at least 92% of the available sequence data for all isolates.** **The core SNP density, representing the average number of SNPs per base pair in the core genome, was 5379 SNPs across 4062284 base pairs in the reference genome. The reference genome used was from CR*Ab* ST2 clone 1 (FJ16), isolated from an adult ICU patient at the CWMH in 2019** (Baleivanualala et al., 2023)**. The tree was rooted using the earliest isolate, from a NICU patient of CWMH isolated in 2016. Isolation dates and hospitals and clonal types are annotated on the tree. The scale bar represents the frequency of mutations per site.**
3. **Core genome SNP phylogeny of clinical and environmental carbapenem resistant *A. baumannii* ST2 clone 3 isolates from of CWMH, LTKH, and LBSH (2020 - 2022). Phylogenetic tree inferred from core genome SNPs of 58 CR*Ab* ST2 clone 3 isolates obtained from patients and high touch surfaces and equipment. The core genome was generated by aligning at least 95% of the available sequence data for all isolates. The core SNP density, representing the average number of SNPs per base pair in the core genome, was 24 SNPs across 4080253 base pairs in the reference genome. The reference genome used was from CR*Ab* ST2 clone 3 (FJ104) isolated from an adult ICU patient at the CWMH in 2020** (Baleivanualala et al., 2024)**. Isolation dates and hospitals are annotated on the tree. The scale bar represents the frequency of mutations per site.**

**Supplementary Figure 3. Core genome SNP phylogeny of environmental and clinical carbapenem resistant *Acinetobacter baumannii* ST25 isolates from Fiji’s CWMH (2021)**

**a.**


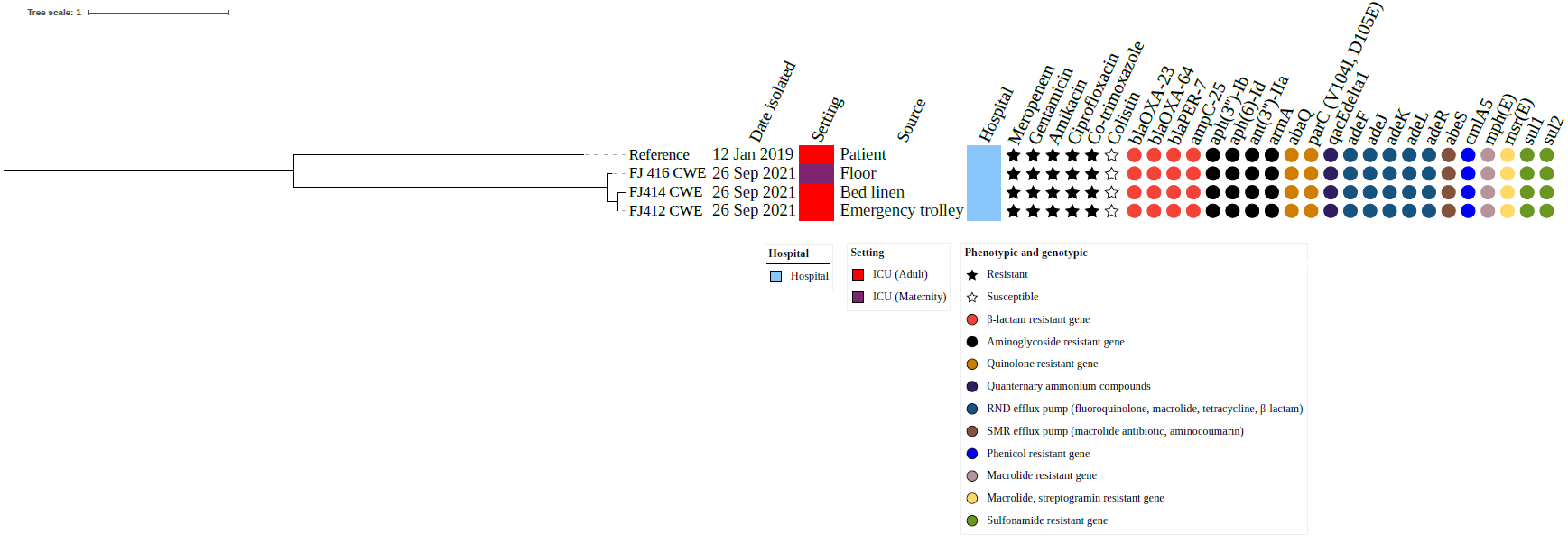


**b.**


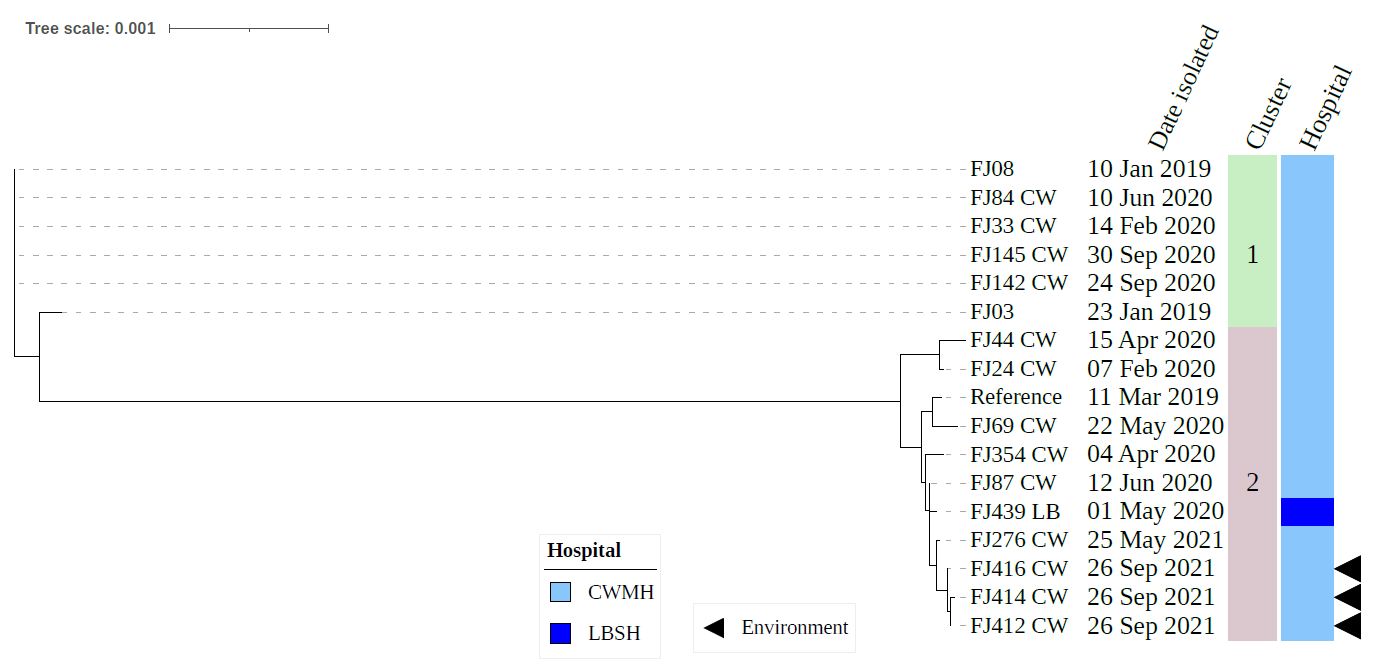


**a****) Core genome SNP phylogeny of environmental carbapenem resistant *A. baumannii* ST25 isolates from CWMH (2021). Phylogenetic tree inferred from core genome SNPs of three CR*Ab* ST25 isolates. The core genome was generated by aligning at least 94% of the available sequence data for all isolates. The core SNP density, representing the average number of SNPs per base pair in the core genome, was 32 SNPs across 4210148 base pairs in the reference genome. The reference genome used was from CR*Ab* ST25 (FJ14), isolated from an adult ICU patient at the CWMH in 2019** (Baleivanualala et al., 2023)**. The tree was rooted using the earliest isolate (reference genome, FJ14). Isolation dates, locations, collection site, and AMR phenotypic and genotypic profiles are annotated on the tree. The scale bar represents the frequency of mutations per site.**

**b. Core genome SNP phylogeny of environmental and clinical carbapenem resistant *A. baumannii* ST25 isolates from CWMH and LBSH (2019 - 2021). Phylogenetic tree inferred from core genome SNPs of 17 CR*Ab* ST25 isolates. The core genome was generated by aligning at least 91% of the available sequence data for all isolates. The core SNP density, representing the average number of SNPs per base pair in the core genome, was 8918 SNPs across 4210148 base pairs in the reference genome. The reference genome used was from CR*Ab* ST25 (FJ14), isolated from an adult ICU patient at the CWMH in 2019** (Baleivanualala et al., 2023)**. The tree was rooted using the earliest isolate (reference genome, FJ14) from CWMH patient. Isolation dates and hospitals are annotated on the tree. The scale bar represents the frequency of mutations per site.**

**Supplementary Figure 4. Core genome SNP phylogeny of environmental and clinical carbapenem resistant *Acinetobacter baumannii* ST499 isolates from Fiji’s CWMH and LTKH (2022)**


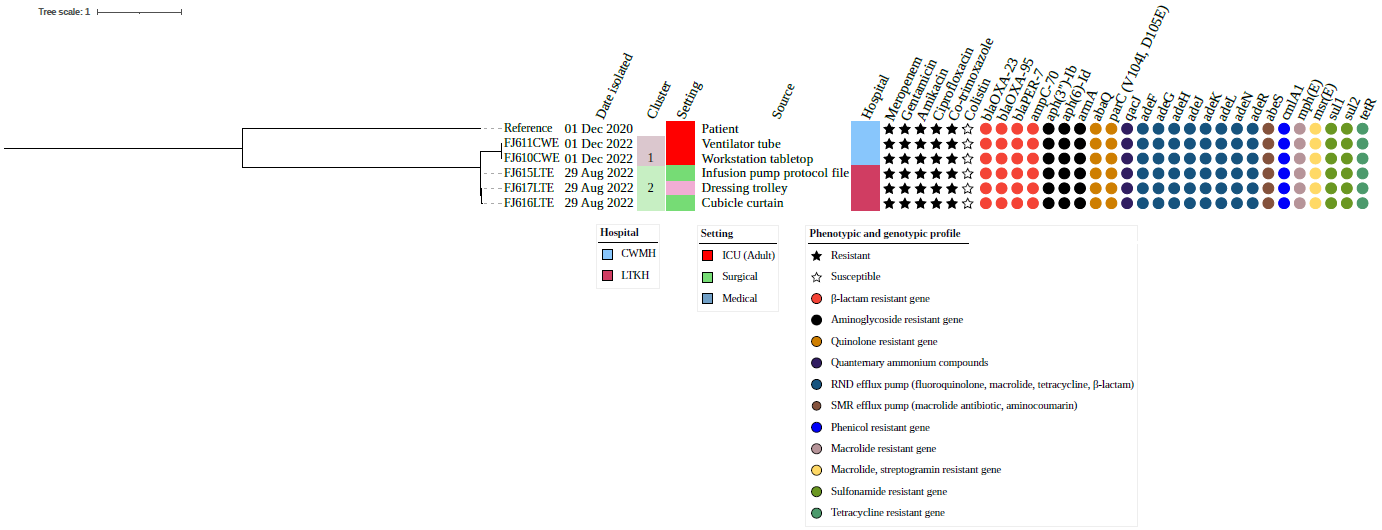


**Core genome SNP phylogeny of environmental carbapenem resistant *A. baumannii* ST499 isolates from CWMH and LTKH (2022). Phylogenetic tree inferred from core genome SNPs of five CR*Ab* ST499 isolates. The core genome was generated by aligning at least 94% of the available sequence data for all isolates. The core SNP density, representing the average number of SNPs per base pair in the core genome, was 2945 SNPs across 4274662 base pairs in the reference genome. The reference genome used was CR*Ab* ST499 (FJ275_CW), isolated from a patient in CWMH in 2020** (Baleivanualala et al., 2024)**. The tree was rooted using the earliest isolate (reference genome, FJ275_CW). Isolation dates, locations, collection site, and AMR phenotypic and genotypic profiles are annotated on the tree. The scale bar represents the frequency of mutations per site.**

**References**

Alcock, B. P., Raphenya, A. R., Lau, T. T. Y., Tsang, K. K., Bouchard, M., Edalatmand, A., Huynh, W., Nguyen, A. L. V., Cheng, A. A., Liu, S., Min, S. Y., Miroshnichenko, A., Tran, H. K., Werfalli, R. E., Nasir, J. A., Oloni, M., Speicher, D. J., Florescu, A., Singh, B., … McArthur, A. G. (2020). CARD 2020: antibiotic resistome surveillance with the comprehensive antibiotic resistance database. *Nucleic Acids Research*, *48*(D1), D517–D525. https://doi.org/10.1093/NAR/GKZ935

Baleivanualala, S. C., Isaia, L., Devi, S. V, Howden, B., Gorrie, C. L., & Matanitobua, S. (2023). Articles Molecular and clinical epidemiology of carbapenem resistant Acinetobacter baumannii ST2 in Oceania : a multicountry cohort study. *The Lancet Regional Health - Western Pacific*, 100896. https://doi.org/10.1016/j.lanwpc.2023.100896

Baleivanualala, S. C., Matanitobua, S., Soqo, V., Smita, S., Limaono, J., Sharma, S. C., Devi, S. V., Boseiwaqa, L. V., Vera, N., Kumar, S., Lalibuli, A., Mailulu, J., Wilson, D., Samisoni, Y., Crump, J. A., & Ussher, J. E. (2024). Molecular and clinical epidemiology of carbapenem resistant Acinetobacter baumannii, Pseudomonas aeruginosa and Enterobacterales in Fiji: a multicentre prospective observational study. *The Lancet Regional Health - Western Pacific*, *47*, 101095. https://doi.org/10.1016/j.lanwpc.2024.101095

Croucher, N. J., Page, A. J., Connor, T. R., Delaney, A. J., Keane, J. A., Bentley, S. D., Parkhill, J., & Harris, S. R. (2015). Rapid phylogenetic analysis of large samples of recombinant bacterial whole genome sequences using Gubbins. *Nucleic Acids Research*, *43*(3), e15. https://doi.org/10.1093/nar/gku1196

Kwan K, Cooper M, La Duc MT, Vaishampayan P, Stam C, Benardini JN, Scalzi G, Moissl-Eichinger C, V. K. (2011). Evaluation of procedures for the collection, processing, and analysis of biomolecules from low-biomass surfaces. *Applied and Environmental Microbiology*, *77*(9), 2943-2953. https://doi.org/10.1128/AEM.02978-10

Larsen, Mette V. Cosentino, Salvatore Rasmussen, S., Friis, Carsten Hasman, Henrik Marvig, R. L., & Jelsbak, Lars Sicheritz-Pontén, Thomas Ussery, David W. Aarestrup, Frank M. Lund, O. (2012). Multilocus sequence typing of total-genome-sequenced bacteria. *Journal of Clinical Microbiology*, *50*(4), 1355-1361. https://doi.org/10.1128/JCM.06094-11

Larsen, M. V., Cosentino, S., Rasmussen, S., Friis, C., Hasman, H., Marvig, R. L., Jelsbak, L., Sicheritz-Pontén, T., Ussery, D. W., Aarestrup, F. M., & Lund, O. (2012). Multilocus Sequence Typing of Total-Genome-Sequenced Bacteria. *Journal of Clinical Microbiology*, *50*(4), 1355. https://doi.org/10.1128/JCM.06094-11

Nguyen Vu, Thao Byun, Jung Hyun D’Souza, Roshan Pinto, Naina Adren Nguyen, Le Phuong Yong, Dongeun Chong, Y. (2020). Adjustment of Modified Carbapenem Inactivation Method Conditions for Rapid Detection of Carbapenemase-Producing Acinetobacter baumannii. *Annals of Laboratory Medicine*, *40*(1), 21-26. https://doi.org/10.3343/alm.2020.40.1.21

Oxford Nanopore Technologies. (n.d.). *Community - Protocol - Rapid sequencing gDNA - barcoding (SQK-RBK004)*. Retrieved 21 June 2022, from https://community.nanoporetech.com/docs/prepare/library_prep_protocols/rapid-barcoding-sequencing-sqk-rbk004/v/rbk_9054_v2_revac_14aug2019

Page, A. J., Taylor, B., Delaney, A. J., Soares, J., Seemann, T., Keane, J. A., & Harris, S. R. (2016). SNP-sites: rapid efficient extraction of SNPs from multi-FASTA alignments. *Microbial Genomics*, *2*(4), e000056. https://doi.org/10.1099/MGEN.0.000056/CITE/REFWORKS

Wang, Y., Zhao, Y., Bollas, A., Wang, Y., & Au, K. F. (2021). Nanopore sequencing technology, bioinformatics and applications. *Nature Biotechnology*, *39*(11), 1348–1365. https://doi.org/10.1038/s41587-021-01108-x

Wick, R. R., Judd, L. M., Gorrie, C. L., & Holt, K. E. (2017). Unicycler: Resolving bacterial genome assemblies from short and long sequencing reads. *PLoS Computational Biology*, *13*(6), 1–22. https://doi.org/10.1371/journal.pcbi.1005595
